# Detection of Antimicrobial Resistance Using Proteomics and the Comprehensive Antibiotic Resistance Database: A Case Study

**DOI:** 10.1002/prca.201800182

**Published:** 2020-02-28

**Authors:** Chih‐yu Chen, Clifford G. Clark, Stacie Langner, David A. Boyd, Amrita Bharat, Stuart J. McCorrister, Andrew G. McArthur, Morag R. Graham, Garrett R. Westmacott, Gary Van Domselaar

**Affiliations:** ^1^ National Microbiology Laboratory Public Health Agency of Canada Winnipeg Manitoba Canada; ^2^ M. G. DeGroote Institute for Infectious Disease Research Department of Biochemistry and Biomedical Sciences DeGroote School of Medicine McMaster University Hamilton Ontario Canada; ^3^ Department of Medical Microbiology and Infectious Diseases Rady Faculty of Health Sciences, Max Rady College of Medicine University of Manitoba Winnipeg Manitoba Canada

**Keywords:** antimicrobial resistance, *Campylobacter jejuni*, Comprehensive Antibiotic Resistance Database, shotgun proteomics, whole genome sequencing

## Abstract

**Purpose:**

Antimicrobial resistance (AMR), especially multidrug resistance, is one of the most serious global threats facing public health. The authors proof‐of‐concept study assessing the suitability of shotgun proteomics as an additional approach to whole‐genome sequencing (WGS) for detecting AMR determinants.

**Experimental Design:**

Previously published shotgun proteomics and WGS data on four isolates of *Campylobacter jejuni* are used to perform AMR detection by searching the Comprehensive Antibiotic Resistance Database, and their detection ability relative to genomics screening and traditional phenotypic testing measured by minimum inhibitory concentration is assessed.

**Results:**

Both genomic and proteomic approaches identify the wild‐type and variant molecular determinants responsible for resistance to tetracycline and ciprofloxacin, in agreement with phenotypic testing. In contrast, the genomic method identifies the presence of the β‐lactamase gene, *bla*_OXA_ *_‐_*_61_, in three isolates. However, its corresponding protein product is detected in only a single isolate, consistent with results obtained from phenotypic testing.

The emergence and spread of antimicrobial resistance (AMR) mechanisms in bacterial pathogens have rendered many antibiotics ineffective, resulting in a global health crisis.^1^ Clinical microbiology laboratories currently perform antimicrobial susceptibility testing (AST) using phenotypic methods, such as the minimum inhibitory concentration (MIC). However, these methods standardly rely on cultured isolates, which can be problematic if the pathogen is slow growing or unculturable. Molecular techniques have been developed to complement traditional culture‐based phenotypic AST. Such techniques include genotypic testing with polymerase chain reaction (PCR), transcriptomic testing with quantitative reverse transcription PCR, and proteomic testing with matrix‐assisted laser desorption/ionization time‐of‐flight mass spectrometry (MALDI‐TOF MS).^2^

Whole genome sequencing (WGS) provides a comprehensive inventory of an organism's functional potential, making it an attractive approach for AST. A number of excellent resources for detecting AMR from WGS data are publicly available.^3^ Of these, the Comprehensive Antibiotic Resistance Database (CARD)^4^ stands out for its high quality, manually curated resistance detection models derived from experimentally verified phenotype–genotype associations reported in the scientific literature, and through collaborations with public health and clinical microbiology laboratories. CARD contains databases specific to the molecular determinants of AMR, including protein homologs, protein and rRNA variants, and regulatory mutations in efflux systems.

Although promising, the suitability of WGS for AST has yet to be established.^5^ Molecular tests that provide evidence of the expression of the implicated genes—such as transcriptomics‐based and proteomics‐based tests—should in theory be superior to genomics‐based tests alone. Transcriptomics approaches to AMR detection are problematic due to their low correlation with protein abundance levels.^6^ Generally, multiple molecular factors contribute to observed discrepancies among gene content, transcriptome, proteome, and phenome. Proteomics methods that directly detect the presence and abundance of the proteins that confer AMR should, in theory, provide the strongest molecular evidence of resistance.^2^

A number of proteomics techniques have been used to study AMR.^7^ Whole‐cell MALDI‐TOF MS has been used successfully for AMR screening, but the resulting fingerprint provides no useful information at the individual protein level. Targeted proteomic approaches, such as those based on selected reaction monitoring, can provide higher sensitivity and specificity for detecting and quantifying specific biomarkers, but these methods require prior knowledge of the target biomarker and are not as suitable for use with the ever‐growing AMR databases. Shotgun proteomics by liquid chromatography‐tandem MS (LC‐MS/MS) provides high‐resolution data that enables investigation of underlying resistance mechanisms as well as specific antimicrobial agent(s) of interest.^8^, ^9^, ^10^, ^11^ Currently, no whole‐proteome‐based AMR detection method yet exists that acts as a functional equivalent to genomic and transcriptomic approaches for surveying an organism's resistome.

As a proof of concept, we examined the potential of applying LC‐MS/MS proteomics data for simultaneous, database‐directed screening of AMR molecular determinants to detect the resistomes in four *Campylobacter jejuni* isolates. We employed the protein homolog and protein variant databases of resistance genes, their products, and associated phenotypes from CARD (https://card.mcmaster.ca).^4^ We compared proteomic AMR detection results to WGS data using previously published proteomic and genomic datasets of our *C. jejuni* isolates,^12^ and assessed them against MIC phenotype—the current gold standard for AST (workflow in Figure S1 and detailed methods, Supporting Information).

Bacteria acquire resistance through two main evolutionary mechanisms: 1) horizontal gene transfer between organisms, and 2) clonal transmission of spontaneously generated mutations in AMR genes. Resistance measures can be qualitative (i.e., presence/absence) or quantitative. We performed quantitative resistance measurement by MIC (**Table** 1) using Sensititre broth microdilution with the CAMPY panel, and interpretations following the Clinical & Laboratory Standards Institute (CLSI) M45 guidelines^13^ and National Antimicrobial Resistance Monitoring System (NARMS) guidelines. Phenotypic testing of isolate 00‐1597 reported resistance to ciprofloxacin, a fluoroquinolone, as well as nalidixic acid. Tetracycline resistance was reported for isolates 00‐0949 and 01‐1512. Since a β‐lactamase was detected in both proteomic and genomic surveys, we also conducted confirmatory testing for β‐lactam MIC by ETest despite a lack of established breakpoint in standard AST for *Campylobacter*. The MIC of ampicillin, a β‐lactam, was greatly elevated in isolate 01‐1512 at levels 32‐fold to 64‐fold higher than our other isolates.

**Table 1:** MIC of *Campylobacter jejuni* isolates

Antimicrobials	Method	MIC [μg mL^−1^]			
		00‐1597	00‐6200	00‐0949	01‐1512
Azithromycin^a^	CAMPY panel (Trek)	0.06	0.06	0.06	0.06
Ciprofloxacin^b^		**16**	0.06	0.06	0.06
Clindamycin^a^		0.5	0.12	0.12	0.12
Erythromycin^b^		1	0.5	0.5	0.5
Florfenicol^a^		2	1	1	1
Gentamicin^a^		1	0.5	0.5	0.5
Nalidixic acid^a^		> **64**	≤4	≤4	≤4
Telithromycin^a^		1	2	0.5	0.5
Tetracycline^b^		0.5	0.12	> **64**	**64**
Ampicillin	ETest	1.5	1	2	64^c^
Amox/clav		0.75	0.75	1	4

*C. jejuni* isolates categorized as phenotypically resistant are bold and underlined.

a NARMS interpretation.

b CLSI M45 interpretation.

c MIC > 30‐fold increase.

**Clinical Relevance**

In our proof‐of‐concept study, proteomic methods are comparable to, and in certain cases, better than genomics methods for AMR detection, as judged by phenotypic testing. The result underscores the value of a large‐scale evaluation of proteomic testing as an additional approach to genomic AMR detection.

We then performed AMR detection from WGS data^12^ using CARD's Resistance Gene Identifier (RGI) software v4.1.0^4^ and ResFinder v3.1.^14^ Both approaches identified the presence of AMR‐associated genes and an allelic variant in various subsets of isolates (**Figure** 1A and Figure S2, Supporting Information). The detected AMR profiles for each isolate largely agreed with the MIC results. The *gyrA* mutant T86I—a target mutation detected in isolate 00‐1597—was associated with ciprofloxacin resistance. The presence of *tetO*, detected in one of two multicopy plasmids in two isolates (00‐0949, 01‐1512) with 100% sequence identity, agreed with MIC for tetracycline resistance.

**Figure 1:**
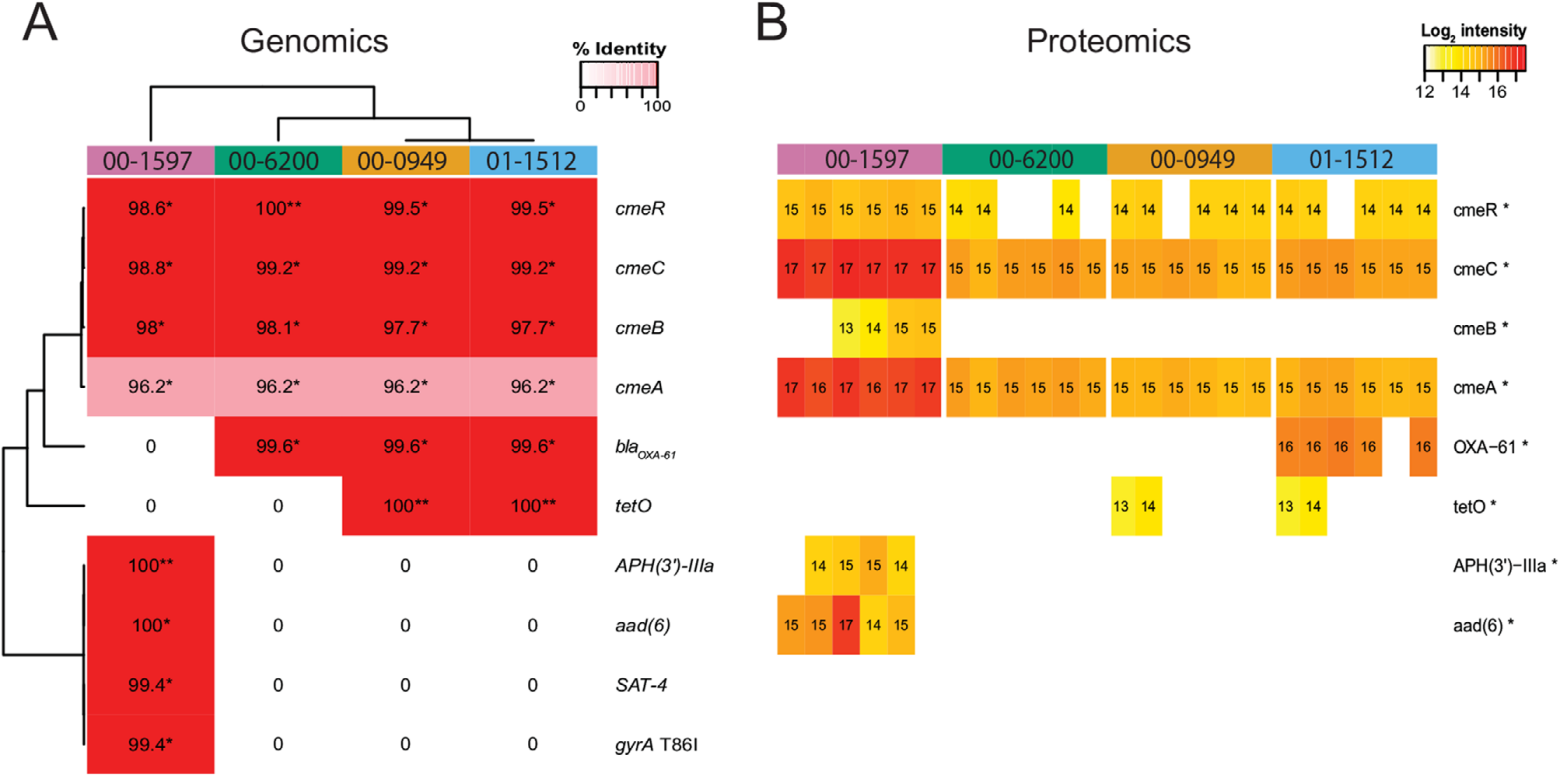
Genomic and proteomic searches of CARD for AMR screening. A) Heatmap of AMR protein sequences identified in WGS data of *Campylobacter* isolates using RGI v4.0.1. Each cell is labeled and shaded from red to pink representing their percent identity match to CARD as indicated in the legend. Only perfect (**) and strict (*) matches as reported by RGI are coloured; entries without a protein sequence match in an isolate are uncoloured. B) Heatmap of AMR protein abundance detected using FDR = 0.01 and an additional presence cut‐off in isolates with six biological replicates. The log_2_ relative intensities are labeled in each cell and colored in a gradient from red to yellow indicating higher and lower abundance, respectively. Proteins with significant abundance variation among groups as tested with ANOVA (Benjamini–Hochberg adjusted *p* ≤ 0.05) are marked with an asterisk.

There were some discrepancies between genomic detections, and in one instance, these detections were also inconsistent with the MIC results. Despite the detection of β‐lactamase genes *bla*_OXA_ *_‐_*_61_ (RGI) or *bla*_OXA_ *_‐_*_450_ (ResFinder) in three isolates, only one of these (01‐1512) reported an elevated MIC value for ampicillin. Furthermore, in isolate 00‐1597, RGI reported the presence of aminoglycoside‐associated genes *APH(*3ʹ*)‐IIIa* and *aad(*6), as well as nucleoside‐associated gene *SAT‐*4, whereas ResFinder reported only the *APH(*3 ʹ*)‐IIIa* gene. The inconsistency is due to both biocuration and bioinformatics: Resfinder's database contains the *aad(*6) sequence, labeled as *ant(*6*)‐Ia*, but does not contain the *SAT‐*4 sequence. Yet these three genes, all contained within one contig, are known plasmid‐associated AMR determinants.^4^ Since our phenotypic assay was limited to the CAMPY panel, only one aminoglycoside agent (gentamicin) was tested and showed susceptibility for all isolates. *APH(*3 ʹ*)‐IIIa* and *aad(*6) are known to confer resistance to aminoglycoside agents other than gentamicin.^15^ Lastly, sequence identities of detected *Campylobacter* multidrug efflux (cme) gene products reported from RGI were more than 96% in all four isolates, whereas ResFinder does not report efflux proteins not associated with plasmids. These Cme‐family proteins, with resistance to multiple antimicrobials, challenged the AMR detection interpretation.^16^ The relative contribution of efflux to overall MIC, whether additive or synergistic, is rarely clear unless known overexpression mutations exist. CARD is actively working on algorithms to predict efflux overexpression via mutation in regulatory proteins or their binding sites (McArthur; personal communication).

We next evaluated AMR detection for each of our *C. jejuni* isolates using previously published quantitative comparative proteomics data from iTRAQ labeling.^12^ Each isolate has six biological replicates. We conducted searches on CARD and Swiss‐Prot databases using MaxQuant v1.6.0.1 (false discovery rates ≤ 0.01 for peptide and protein levels).^17^ We used MaxQuant‐reported abundance for statistical differential abundance analyses. We compared relative protein abundance levels across isolates for each AMR determinant detected from CARD using one‐way Analysis of Variance (ANOVA) with Benjamini–Hochberg correction and found significantly different abundances in at least one isolate, for all AMR determinants (Figure S3, Supporting Information). *TetO* had significantly higher protein abundances in isolates 00‐0949 and 01‐1512 (≥ eightfold; one‐sided *t*‐test *p*‐value = 1.3 × 10^−12^), which was consistent with our genomic and MIC results for tetracycline resistance. To reduce false positives and filter out abundance from baseline noise, an additional abundance cut off was implemented to determine protein presence using the 95^th^ percentile abundance of reversed/decoy proteins (method in Supporting Information). With the higher level of stringency, a subset of proteins was not unanimously detected as present in all six biological replicates (Figure 1B). The variation between biological replications as well as analytical thresholds likely contributed to variability of abundance and protein presence among replicates (further discussion in Supporting Information). Lastly, while we do not have MIC results for the corresponding antibiotics, *APH*(3ʹ)‐*IIIa* and *aad*(6) proteins were detected for isolate 00‐1597, consistent with genomic results from RGI, whereas *SAT*‐4 protein was not detected.

The quantitative proteomics data also revealed protein abundance variation for several gene products that were present in multiple isolates (Figure 1B and Figure S3, Supporting Information). Despite gene presence in all four isolates, the Cme‐family proteins had significantly higher protein abundance in only isolate 00‐1597 compared to all others (maximum one‐sided *t*‐test *p*‐value = 5.4 × 10^−4^). Interestingly, despite gene presence detected in three isolates, the significantly higher OXA‐61 protein abundance level in isolate 01‐1512 (maximum one‐sided *t*‐test *p*‐value = 1.2 × 10^−15^) was consistent with elevated MIC to ampicillin. Higher β‐lactam MIC levels were reported to be dependent on expression levels of various secreted β‐lactamases.^2^ A guanine‐to‐thymine mutant in the *bla*_OXA_ *_‐_*_61_ promoter has been shown emperically to create a new TATA box, raise OXA‐61 protein levels, and elevate MICs to ampicillin.^18^ Upon examination of *bla*_OXA‐61_ promoter region, isolate 01‐1512 indeed encoded thymine at this position, whereas the other two isolates harbored the wild‐type guanine (Figure S4, Supporting Information). Neither CARD nor ResFinder currently contain information on this (or any other) promoter mutation, which led to false‐positive genomic ampicillin resistance predictions for isolates 00‐0949 and 00‐6200, whereas proteomics results were consistent with observed ampicillin MIC despite the missing database information.

To detect AMR‐associated variants on proteomics data using CARD, we examined GyrA peptides. **Figure** 2A highlighted five GyrA peptides, of which two peptide‐pairs had a single amino acid mutation, and one peptide was conserved. Figure 2B showed their reported peptide abundance levels from MaxQuant with our additional peptide‐length dependent cut offs, as described in Supporting Information. The conserved peptide (“IMAIIPTTDFDESK”), found across all isolates, had no significant difference in relative abundance (one‐way ANOVA *p*‐value = 0.42; Figure 2B). This observation is consistent with this region of the GyrA being conserved across all the isolates, which is confirmed by the genomic data (Figure 2A). In contrast, regions harboring variation across isolates are expected to give rise to differential peptide abundances, which is borne out by our results. The AMR‐associated GyrA T86I mutant resulted in a distinct spectrum due to the mass differences between threonine and isoleucine, whereas the E393K mutant resulted in a shorter tryptic‐digested peptide (Figure 2A). The variation between biological replications and analytical approaches mentioned previously likely contributed to variability of abundance and peptide presence among replicates. Despite only being detectable in three of six biological replicates, the abundance of the peptides with T86I and E393K mutations averaged 13‐fold higher in isolate 00‐1597 than others (one‐sided *t*‐test *p*‐values = 0.0013 and 0.0010, respectively; Figure 2B and Figure S5B, Supporting Information). In cases where resistance results from amino acid mutations, a peptide‐centric approach will be more appropriate for detecting AMR than the corresponding protein‐centric approach. However, peptide‐centric analyses can suffer near the technical limit of detection, especially since variant identification fully depends on the abundance of the peptide harboring resistance variants. Despite this limitation, our findings demonstrated the capacity of proteomics data to inform accurately on the presence and abundance of protein variants in isolate 00‐1597, consistent with ciprofloxacin MIC and independent of WGS data.

**Figure 2:**
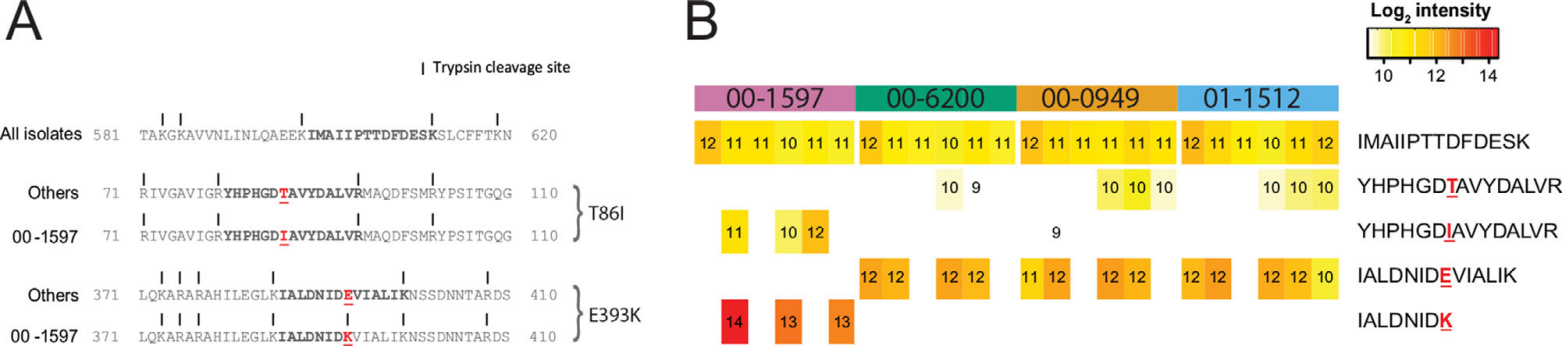
Conserved peptide and peptide variants of GyrA and their relative peptide abundance in isolates. The three examples provided show a peptide shared among isolates, and two wild type/mutant peptide‐pairs. A) Examples of peptide sequences appear in bold with the mutations in red and underlined. All identified mutations are harbored by the 00‐1597 isolate; all other isolates are wild type using the Swiss‐Prot database as a reference. B) Heatmap of peptide abundance using FDR = 0.01 and an additional length‐dependent presence cut off in isolates with six biological replicates. The log_2_ relative peptide abundance is labeled and the shading from red to yellow indicates higher and lower abundance, respectively. Peptides that are undetected or do not pass the additional presence cut off are uncolored.

Unlike most existing studies that focus on resistance mechanisms using high‐resolution MS proteomics datasets,^19^, ^20^ our study instead focused on evaluating a proteome‐wide approach for AMR detection using CARD. The findings highlighted capacity for proteome‐based AMR screening through detection of wild‐type and variant molecular determinants, independent of WGS data. Lastly, while no clinically relevant breakpoint determination currently exists for ampicillin for *Campylobacter* owing to lack of its clinical usage, the concordance between ampicillin MIC and OXA‐61 protein abundance levels still suggests an added value of proteomics compared to genomics when detecting AMR determinants and interpreting phenotype.

This case study involving four *C. jejuni* isolates serves as proof of concept for the utility of proteomics for AMR detection. Yet to fully evaluate the capability and accuracy for proteomics‐based AMR screening, additional analysis of proteomics data from a larger number of isolates will be needed. Furthermore, both genomic/proteomic analyses are based on CARD and thus are dependent on its comprehensiveness. Continued growth and refinement of AMR databases will be needed to reduce false positive and false negative detection rates for genomic and proteomic analyses, with the observed *bla*_OXA_ *_‐_*_61_ promoter mutation being one such excellent example.

In summary, although phenotypic analysis remains the current gold standard for AMR testing and WGS approach is faster without the need for culturing, our approach (generating proteomics data de novo and leveraging CARD's curated data) appears beneficial for simultaneous antimicrobial multidrug resistance inference, offers potential benefits over in silico AMR genomic detection, and—with further development and larger sample size—may enrich current AMR phenotypic assessment.

Proteomic and WGS datasets previously published are publicly available.^12^ Analytical R scripts and processed datasets for the manuscript can be accessed on GitHub (https://github.com/phac-nml/proteomics4AMR).

## Conflict of Interest

The authors declare no conflict of interest.

## Supporting information

Supporting Information

## References

[1] World Health Organization , Antimicrobial Resistance: Global Report on Surveillance 2014, https://www.who.int/drugresistance/documents/surveillancereport/en/.

[2] A. C. Fluit , M. R. Visser , F. J. Schmitz , Clin. Microbiol. Rev. 2001, 14, 836. 11585788 10.1128/CMR.14.4.836-871.2001 PMC89006

[3] A. G. McArthur , K. K. Tsang , Ann. N. Y. Acad. Sci. 2017, 1388, 78. 27875856 10.1111/nyas.13289

[4] B. Jia , A. R. Raphenya , B. Alcock , N. Waglechner , P. Guo , K. K. Tsang , B. A. Lago , B. M. Dave , S. Pereira , A. N. Sharma , S. Doshi , M. Courtot , R. Lo , L. E. Williams , J. G. Frye , T. Elsayegh , D. Sardar , E. L. Westman , A. C. Pawlowski , T. A. Johnson , F. S. L. Brinkman , G. D. Wright , A. G. McArthur , Nucleic Acids Res. 2017, 45, D566. 27789705 10.1093/nar/gkw1004 PMC5210516

[5] M. J. Ellington , O. Ekelund , F. M. Aarestrup , R. Canton , M. Doumith , C. Giske , H. Grundman , H. Hasman , M. T. G. Holden , K. L. Hopkins , J. Iredell , G. Kahlmeter , C. U. Köser , A. MacGowan , D. Mevius , M. Mulvey , T. Naas , T. Peto , J. M. Rolain , N. W, Samuelsen , Clin. Microbiol. Infect. 2017, 23, 2.[CrossRef] 27890457 10.1016/j.cmi.2016.11.012

[6] C. Vogel , E. M. Marcotte , Nat. Publ. Gr. 2012, 13, 227. 10.1038/nrg3185 PMC3654667 22411467

[7] Y. Charretier , J. Schrenzel , Proteomics ‐ Clin. Appl. 2016, 10, 964. 27312049 10.1002/prca.201600041

[8] Y. Takebayashi , W. A. K. W. N. Ismah , J. Findlay , K. J. Heesom , J. Zhang , O. M. Williams , A. P. MacGowan , M. B. Avison , bioRxiv 2017, 10.1101/138594.

[9] Y. Charretier , T. , T. Cecchini , C. Bardet , A. Cherkaoui , C. Llanes , P. Bogaerts , S. Chatellier , J. ‐P. Charrier , J. Schrenzel , Front. Microbiol. 2015, 6, 81. 25713571 10.3389/fmicb.2015.00081 PMC4322712

[10] T. Cecchini , E.J. Yoon , Y. Charretier , C. Bardet , C. Beaulieu , X. Lacoux , J. ‐D. Docquier , J. Lemoine , P. Courvalin , C. Grillot‐Courvalin , J.P. Charrier , Mol. Cell. Proteomics 2018, 17, 442. 29259044 10.1074/mcp.RA117.000107 PMC5836370

[11] W. A. K. Wan Nur Ismah , Y. Takebayashi , J. Findlay , K. J. Heesom , J.C. Jiménez‐Castellanos , J. Zhang , L. Graham , K. Bowker , O. M. Williams , A. P. MacGowan , M. B. Avison , Antimicrob. Agents Chemother. 2018, 62, e01814. 29263066 10.1128/AAC.01814-17 PMC5826109

[12] C. G. Clark , C. yu Chen , C. Berry , M. Walker , S. J. McCorrister , P. M. Chong , G. R. Westmacott , PLoS One, 2018, 13, e0190836. 29293692 10.1371/journal.pone.0190836 PMC5749857

[13] CLSI , M45: Methods for Antimicrobial Dilution and Disk Susceptibility Testing of Infrequently Isolated or Fastidious Bacteria, 3rd ed., CLSI, Wayne, PA 2016.

[14] E. Zankari , H. Hasman , S. Cosentino , M. Vestergaard , S. Rasmussen , O. Lund , F. M. Aarestrup , M. V. Larsen , J. Antimicrob. Chemother. 2012, 67, 2640. 22782487 10.1093/jac/dks261 PMC3468078

[15] S. Zhao , G. H. Tyson , Y. Chen , C. Li , S. Mukherjee , S. Young , C. Lam , J. P. Folster , J. M. Whichard , P. F. McDermott , Appl. Environ. Microbiol. 2015, 82, 459. 26519386 10.1128/AEM.02873-15 PMC4711122

[16] N. M. Iovine , Virulence, 2013, 4, 230. 23406779 10.4161/viru.23753 PMC3711981

[17] S. Tyanova , T. Temu , J. Cox Nat. Protoc., 2016, 11, 2301. 27809316 10.1038/nprot.2016.136

[18] X. Zeng , S. Brown , B. Gillespie , J. Lin , J. Antimicrob. Chemother. 2014, 69, 1215. 24408987 10.1093/jac/dkt515

[19] C. R. Lee , J. H. Lee , K. S. Park , B. C. Jeong , S. H. Lee , Front. Microbiol. 2015, 6, 828 10.3389/fmicb.2015.00828. 26322035 PMC4531251

[20] A. J. Park , J. R. Krieger , C. M. Khursigara , FEMS Microbiol. Rev. 2016, 40, 323. 26790948 10.1093/femsre/fuv051

