# Supplementary material for: Detection of Antimicrobial Resistance Using Proteomics and the Comprehensive Antibiotic Resistance Database: A Case Study
Source: Proteomics Clin Appl. 2020 Feb 28;14(4):1800182. doi: 10.1002/prca.201800182 (PMC7378939; doi:10.1002/prca.201800182)
Supplement: Supplementary file 1 — Supporting Information [file PRCA-14-1800182-s001.docx]

# Supplemental Material 1

### Methods

All data analyses unless otherwise stated were conducted using R version 3.3.2.^[1]^

#### MIC

Antibiotic susceptibilities were assessed using the broth microdilution Sensititre™ Automated Microbiology System (Trek Diagnostic Systems Ltd., Oakwood Village, OH, USA) with CAMPY panel (https://www.thermofisher.com/order/catalog/product/CAMPY) according to Clinical and Laboratory Standards Institute (CLSI) M45 guidelines^[2]^ and interpreted using established CLSI breakpoints. As CLSI-approved breakpoints are available only for ciprofloxacin, erythromycin, and tetracycline for *Campylobacter*, others (nalidixic acid, azithromycin, telithromycin, clindamycin, florfenicol and gentamicin) are reported using interpretive criteria harmonized with the National Antimicrobial Resistance Monitoring System (NARMS). Given there are no breakpoints for β-lactams, as a proof of concept, we also explored whether concordance exists between MIC values and detectable protein abundance levels. Antibiotic susceptibilities for β-lactams, ampicillin and amoxicillin-clavulanate, were assessed using ETest® (bioMérieux, Marcy l'Etoile, France).

#### Genomics query

To avoid removal of genes within small contigs, the original FASTQ files for the four isolates^[3]^ were downloaded from NCBI and reanalyzed with SPAdes 3.11.1 tool^[4]^ to obtain contigs with no minimum length cut-off. All contigs were analyzed by the RGI v4.1.0 tool at the CARD website to identify hits to the CARD database of AMR-associated genes and variants.^[5]^ RGI uses Prodigal^[6]^ to predict open reading frames, Diamond^[7]^ to detect homologs and CARD curated bit-score cut-offs to evaluate significance. “Perfect” and “strict” hits were extracted for perfect matches to a known resistance gene in CARD and inexact matches to the gene variant models defined by CARD, respectively. The contig sequences also were searched with the ResFinder 3.1 tool^[8]^ with both ‘Acquired antimicrobial resistance genes’ and ‘chromosomal point mutations’ options selected. The contig sequences as well as results from RGI and ResFinder can be accessed on GitHub (https://github.com/phac-nml/proteomics4AMR)

#### Proteomics query

Isolates for the published proteomics data were trypsin-digested and labelled with isobaric tags for relative and absolute quantitation (iTRAQ) with six replicates.^[3]^ MaxQuant version 1.6.0.1^[9]^ was used to identify and quantify peptides for each isolate. The labeling parameters were set for iTRAQ isobaric tags with the precursor ion fraction quantification filter. The false discovery rates (FDRs) at peptide spectrum match and protein levels were set to the default values of 0.01. Database searches were conducted against CARD version 2.0.0^[5]^ (2239 homolog proteins and 140 proteins with variants) and the Swiss-Prot^[10]^ database for *C*. *jejuni* (1625 proteins). The Swiss-Prot database was used instead of inferred protein sequences from WGS in order for the proteomic determination to be independent of the WGS data. A total of 1436 protein groups were identified by MaxQuant. Median normalization of protein levels was conducted three times between and within runs with additional LOESS normalization. Only proteins with sequence coverage greater than 20% were analyzed further. Log_2_ transformation of the normalized intensity was examined to evaluate inferred AMR peptide and protein abundance in each isolate. The proteomics results can be accessed on GitHub (<https://github.com/phac-nml/proteomics4AMR>).

#### Supplementary discussion on variability of peptide and protein abundance across biological replicates

Peptide and protein abundance variability can result from a combination of biological variation between replicates and technical artefact. Biologically, there can be natural protein abundance variation between the biological replicates. Technologically, not all proteins/peptides present within a sample may be detected due to the stochastic nature of the shotgun proteomics approach, the ionizability of peptide analytes, and the detection limit of the instrumentation. In MaxQuant, identification of peptides is performed through the search of peptide and fragment masses in the database then scored by a probabilistic approach to generate the peptide score. The presence and abundance of each peptide are evaluated by the significance cut-off level (FDRs from target-decoy analysis). Varying the cut-off level can influence the identification call for peptides with borderline scores, such as peptides that are similar, but not strong matches to the sequences in the database. False positives and false negatives can also occur from the abundance computation method. For false positives, the noise may be erroneously detected as a signal above the minimum mass detection limit, whereas for false negatives, the true signal may be too weak to be detected and thus are not reported.

In summary, proteins and peptides at borderline abundance may not be detected depending on other proteins in the sample and analytical approaches as well as significance cut-off levels. The biological and technical variability emphasize the need for replicates to provide more statistical power and confidence in the results. Lastly, we note that without spike-in, the reported abundances are relative, and when considering log_2_ ratios between isolates, the baseline noise is canceled out in isolates.

#### Protein and peptide presence cut-offs

Using the MaxQuant FDR cut-offs, non-zero but consistent baseline levels were reported for TetO in isolates 00-1597 and 00-6200 despite the absence of the *tetO* gene (see for example Figure S3B). Such artefacts leading to false positives may result from baseline noise in peak detection, or from the significance stringency of peptide score for the confidence in matching between spectra and peptides. Due to false positive protein and peptide abundance levels observed where there was no genomic presence, we implemented thresholds to determine presence or absence in addition to the MaxQuant FDR cut-offs. At the protein level, the 95^th^-percentile abundance of reversed proteins was used as the cut-off (log_2_ abundance of 13.76; Figure S3C). At the peptide level, median abundance levels within an assay for each reversed peptides were first compiled and locally-estimated scatterplot smoothing (LOESS; span=1; degree=2) was conducted on the 95^th^-percentile reversed peptide abundance levels across peptide lengths (Figure S5C). The fitted LOESS line was then used as the peptide-length dependant cut-offs to determine peptide presence. The approach was taken due to the general observation of higher peptide abundance detected for smaller peptides as shown in Figure S5C. Figures S3A and S5A with the additional presence/absence filters were reported in the main figure (Figures 1B and 2B), whereas differential abundance analyses were conducted using MaxQuant reported abundance as reported in figures S3B and S5B.

### References

[1] R Core Team, R: A Language and Environment for Statistical Computing. Vienna, Austria, 2016.

[2] CLSI, in Standard: M45; Methods for Antimicrobial Dilution and Disk Susceptibility Testing of Infrequently Isolated or Fastidious Bacteria, Wayne, PA. (M45Ed3EA2E), **2016**; 3rd ed.

[3] C. G. Clark, C. yu Chen, C. Berry, M. Walker, S. J. McCorrister, P. M. Chong, G. R. Westmacott, *PLoS One*, **2018**, DOI:10.1371/journal.pone.0190836.

[4] A. Bankevich, S. Nurk, D. Antipov, A. A. Gurevich, M. Dvorkin, A. S. Kulikov, V. M. Lesin, S. I. Nikolenko, S. Pham, A. D. Prjibelski, A. V. Pyshkin, A. V. Sirotkin, N. Vyahhi, G. Tesler, M. A. Alekseyev, P. A. Pevzner, *J. Comput. Biol.*, **2012**, DOI:10.1089/cmb.2012.0021.

[5] B. Jia, A. R. Raphenya, B. Alcock, N. Waglechner, P. Guo, K. K. Tsang, B. A. Lago, B. M. Dave, S. Pereira, A. N. Sharma, S. Doshi, M. Courtot, R. Lo, L. E. Williams, J. G. Frye, T. Elsayegh, D. Sardar, E. L. Westman, A. C. Pawlowski, T. A. Johnson, F. S. L. Brinkman, G. D. Wright, A. G. McArthur, *Nucleic Acids Res.*, **2017**, DOI:10.1093/nar/gkw1004.

[6] D. Hyatt, G. L. Chen, P. F. LoCascio, M. L. Land, F. W. Larimer, L. J. Hauser, *BMC Bioinformatics*, **2010**, DOI:10.1186/1471-2105-11-119.

[7] B. Buchfink, C. Xie, D. H. Huson, Fast and sensitive protein alignment using DIAMOND, *Nature Methods*. 2014.

[8] E. Zankari, H. Hasman, S. Cosentino, M. Vestergaard, S. Rasmussen, O. Lund, F. M. Aarestrup, M. V. Larsen, *J. Antimicrob. Chemother.*, **2012**, DOI:10.1093/jac/dks261.

[9] S. Tyanova, T. Temu, J. Cox, *Nat. Protoc.*, **2016**, DOI:10.1038/nprot.2016.136.

[10] A. Bairoch, *Nucleic Acids Res.*, **2000**, DOI:10.1093/nar/28.1.45.

[11] X. Zeng, S. Brown, B. Gillespie, J. Lin, *J. Antimicrob. Chemother.*, **2014**, DOI:10.1093/jac/dkt515.

### Supplementary Figures


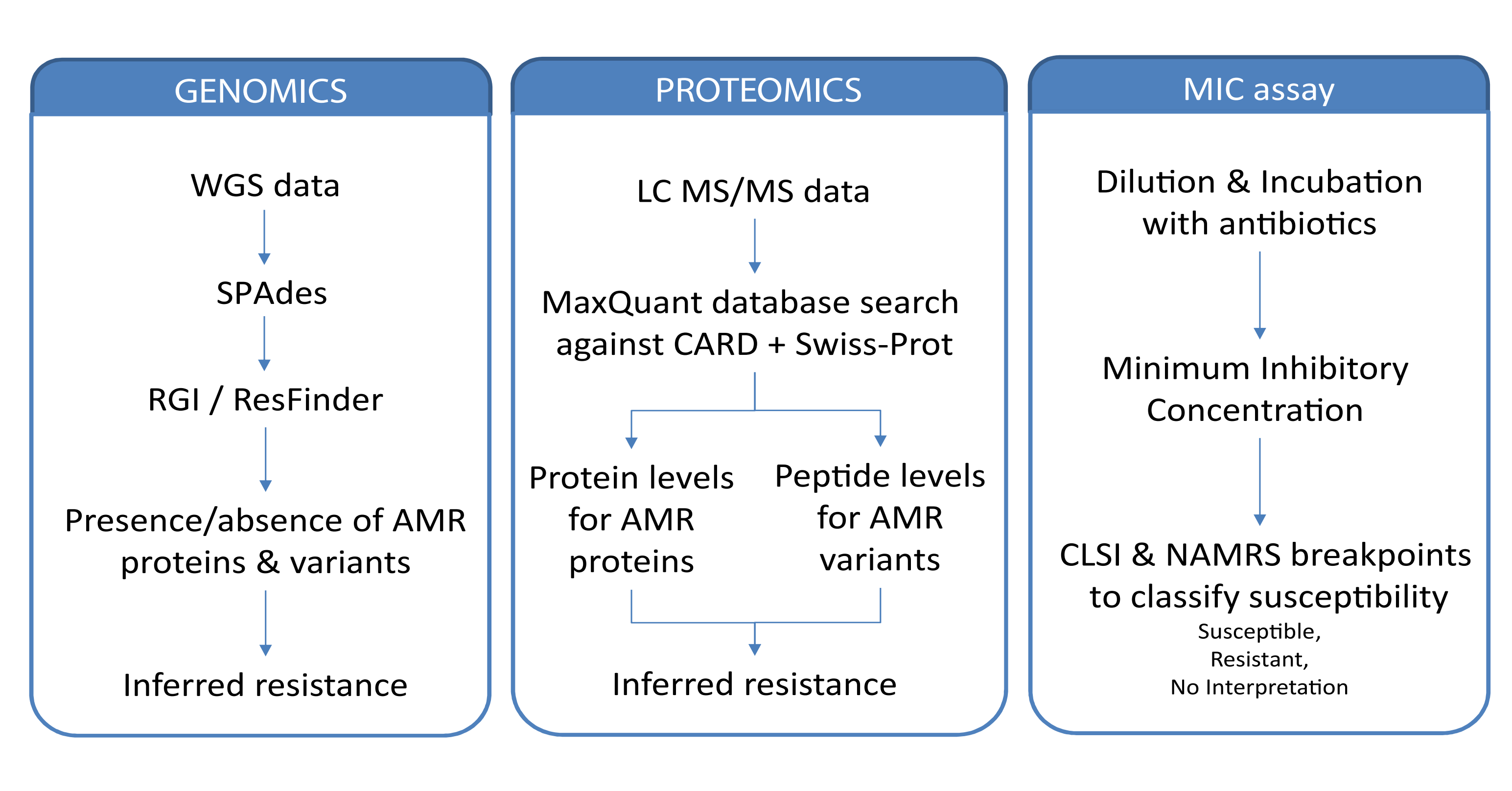


**Figure S1. Workflow of the pilot AMR prediction study through genomics, proteomics and MIC assay.**


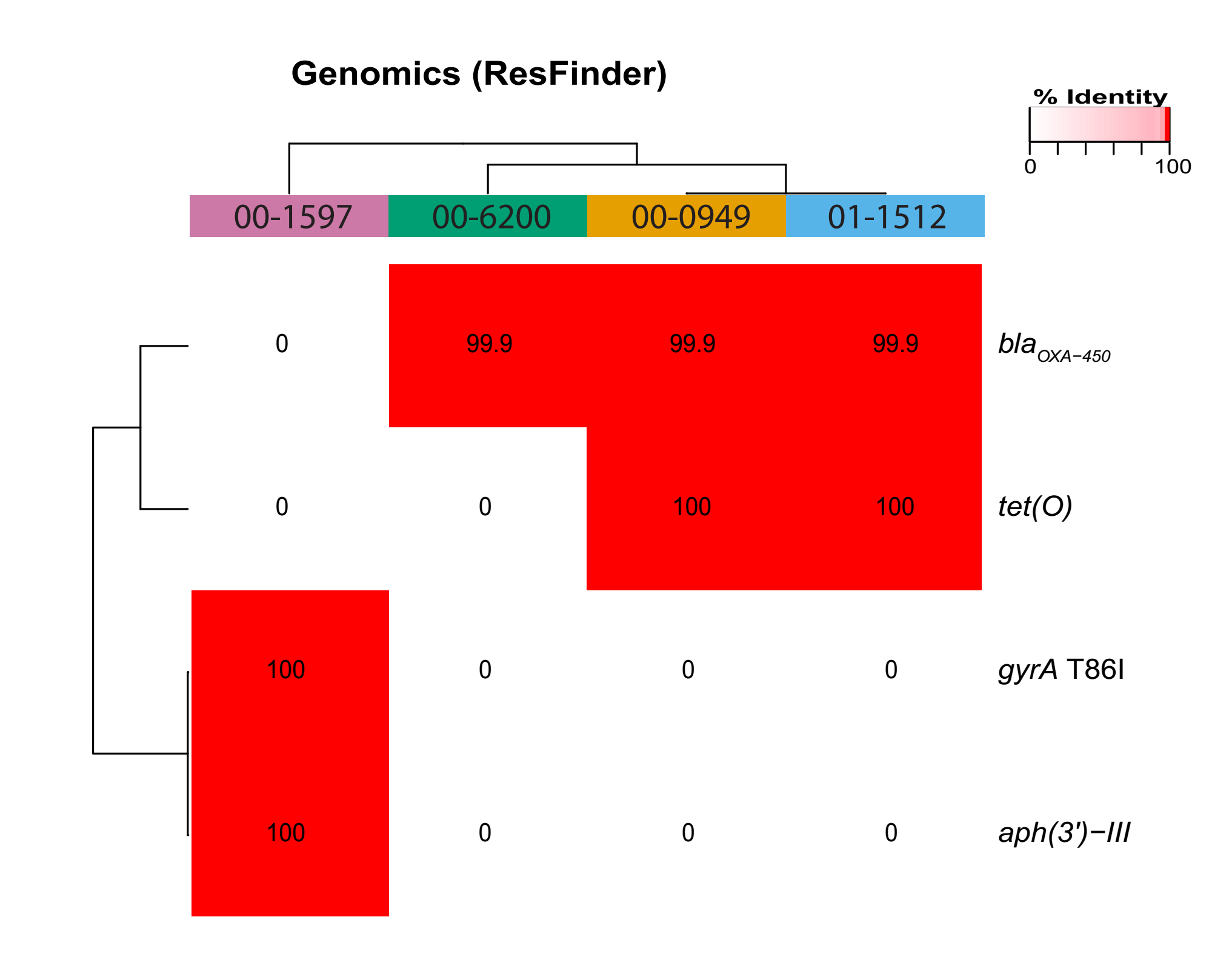


**Figure S2. Genomic query on ResFinder for AMR screening.**Full contig sequences of the four isolates were used as input for the ResFinder 3.1 tool.


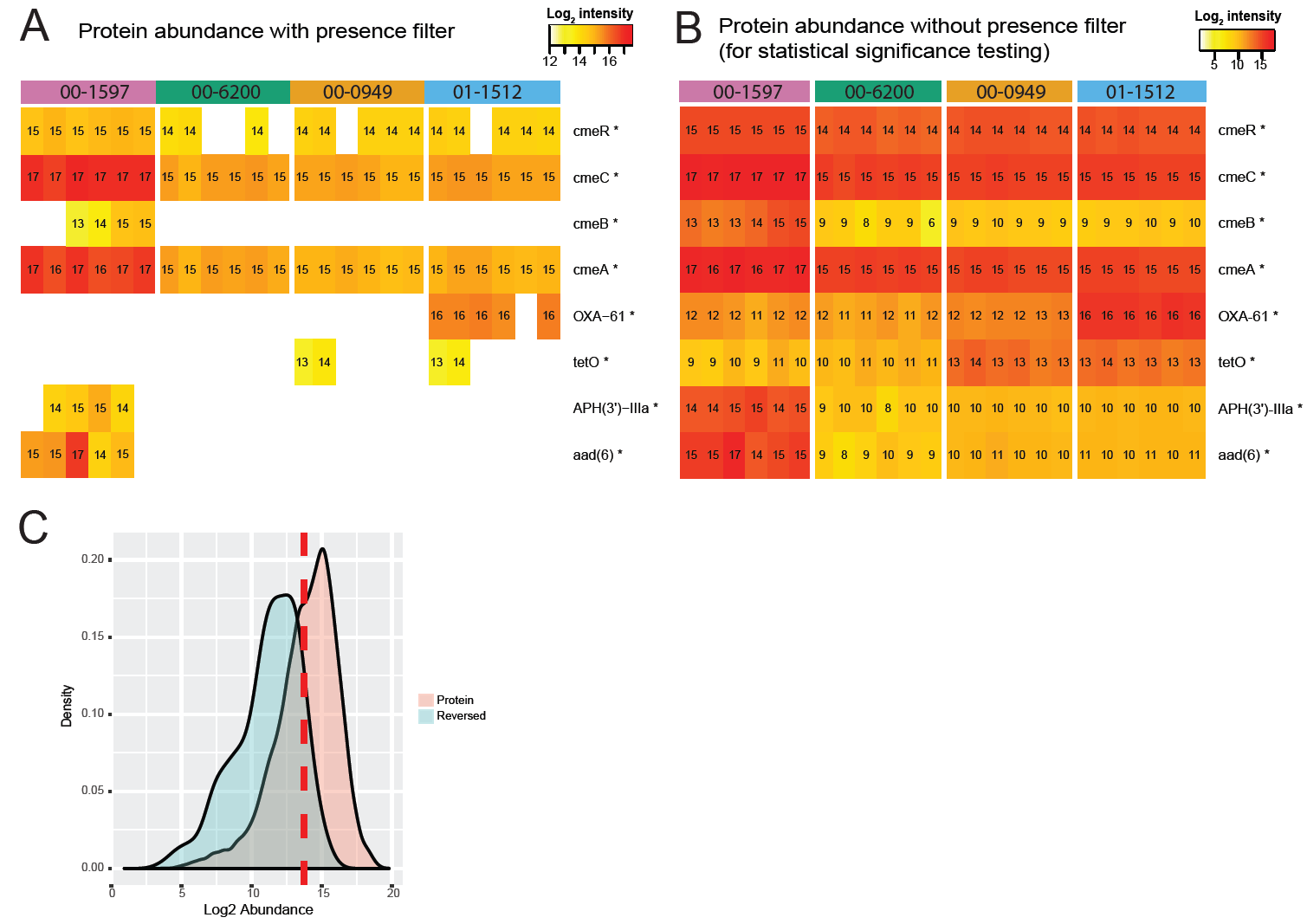


**Figure S3. Protein abundance with and without the additional presence filter.** Heatmaps show the AMR protein abundance detected in isolates with six biological replicates after **A** and before **B** the additional presence cut-off. The log_2_ relative intensities are labeled in each cell and colored in a gradient from red to yellow indicating higher and lower abundance, respectively. Proteins with significant abundance variation among groups as tested with ANOVA (Benjamini-Hochberg adjusted p ≤ 0.05) are marked with an asterisk. **C** The density plot shows the log_2_ abundance distributions of target and reversed proteins. The cut-off for protein presence as determined from the abundance of reversed peptides at 95^th^ percentile is shown with the red dashed line.


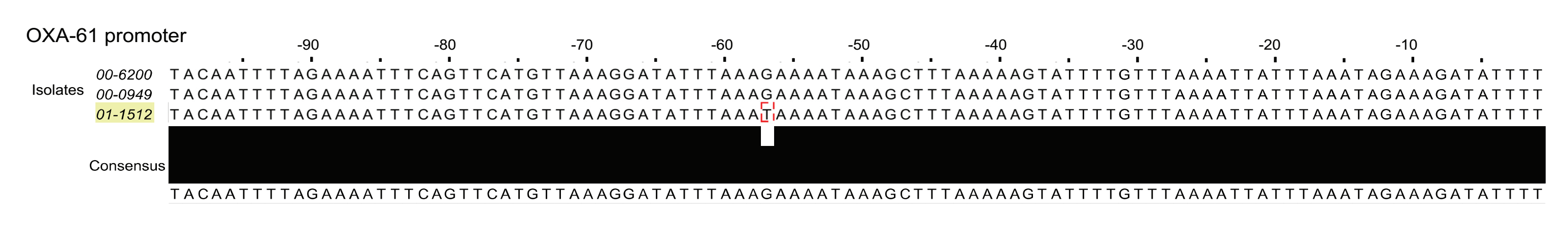


**Figure S4. *bla_OXA-61_* promoter sequence from isolates with the gene.**
The G 🡪T promoter variant at -57 bp upstream of the start codon is only present in isolate 01-1512 and is known to create a TATA box, increasing OXA-61 protein levels and ampicillin resistance in *C. Jejuni.*^[11]^

**
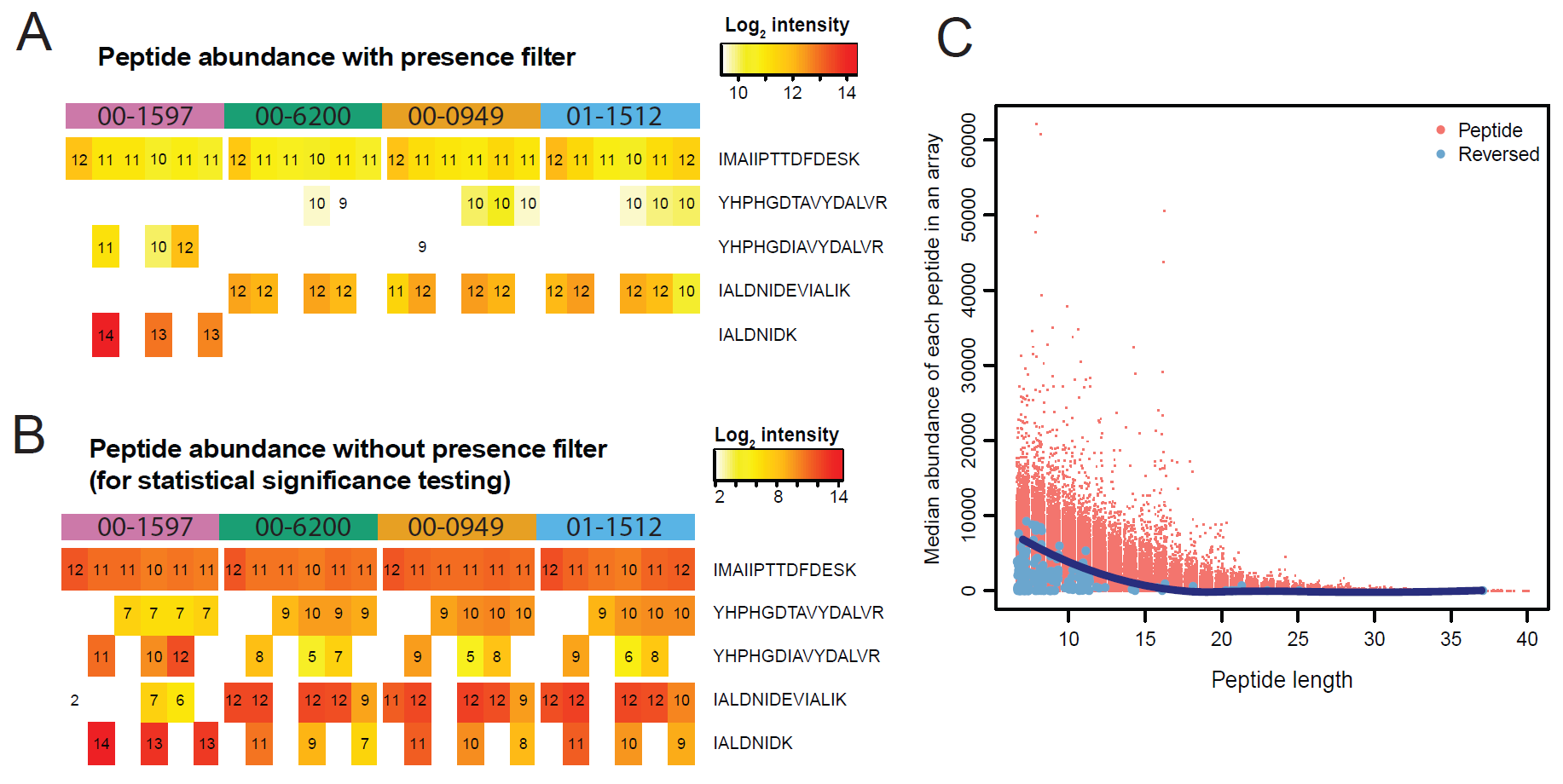
**

**Figure S5. Peptide abundance with and without the presence filter.** Heatmap of peptide abundance in isolates with six biological replicates after **A** and before **B** the additional presence cut-off. The log_2_ relative peptide abundance, is labeled and the shading from red to yellow, indicating indicates higher and lower abundance, respectively. Peptides that are undetected or do not pass the additional presence cut-off are uncolored. **C** The scatter plot shows the median abundance of each target (red) or reversed (blue) peptide with respect to the corresponding peptide length. The LOESS fitted line of the 95^th^-percentile reversed peptide abundance across peptide lengths is shown in dark blue and used as the length dependent cut-offs for presence.
